# Efficacy of Vitamin C Vaginal Tablets as Prophylaxis for Recurrent Bacterial Vaginosis: A Randomised, Double-Blind, Placebo-Controlled Clinical Trial

**DOI:** 10.4021/jocmr1489w

**Published:** 2013-06-21

**Authors:** Vladislav N. Krasnopolsky, Vera N. Prilepskaya, Franco Polatti, Nina V. Zarochentseva, Guldana R. Bayramova, Maurizio Caserini, Renata Palmieri

**Affiliations:** a Research Centre of Obstetrics and Gynaecology of Moscow Region, Moscow, Russia; b Research Centre of Obstetrics, Gynaecology and Perinatology of Rosmedtechnologies, Moscow, Russia; c University of Pavia - “Medicine and Surgery” Faculty, Pavia, Italy; d Scientific Department, Polichem SA, Lugano, Switzerland

**Keywords:** Bacterial vaginosis, Bacterial vaginosis prophylaxis, Vitamin C vaginal tablets, Ascorbic acid vaginal tablets

## Abstract

**Background:**

The aetiology of bacterial vaginosis (BV) is still unclear but it is currently considered to be a synergistic polymicrobial syndrome. BV can often arise as a chronic or recurrent disease. The reason for such recurrences is not well elucidated. Previous studies have suggested that vaginal vitamin C may be a useful treatment in reducing recurrence rate, by increasing vaginal acidification and thereby making up for the decrease in hydrogen peroxide that results from a reduction in the number of lactobacilli present. Based on the above-mentioned consideration, a study was performed that aimed to evaluate the effect of vitamin C in the prophylaxis of BV relapses.

**Methods:**

This was a randomised, double-blind, placebo-controlled, parallel-group clinical trial. One hundred and forty-two women, after having been cured from a recent episode of BV by either metronidazole or clindamycin, were randomised to receive vitamin C (74 patients) or placebo (68 patients) as prophylaxis for six monthly cycles, starting within 24 hours of the determination of ‘BV cure’. The patients applied one vaginal tablet once a day for 6 consecutive days per month after menses.

**Results:**

The rate of BV recurrence during the first 3 months was considerably lower in the vitamin C group (6.8%) than in the placebo (14.7%) group. Considering a 6-month treatment period, the recurrence rate in the vitamin C group (16.2%) was significantly lower (P = 0.024) than in the placebo group (32.4%). Moreover, at the same time point, the survival analysis of Kaplan Meyer was significant in favour of the vitamin C group compared with the placebo group (P = 0.029).

**Conclusions:**

The study showed that regular use of 250 mg ascorbic acid vaginal tablets on 6 days per month for 6 months after successful treatment of bacterial vaginosis halves the risk of recurrence from 32.4% to 16.2% (P = 0.024).

## Introduction

Bacterial vaginosis (BV) is a common condition that affects almost one-third of women of childbearing age (29%) [1]. In Caucasian women, the prevalence is 5% to 15%; in African and black American women, it is 45% to 55%; and in Asian women the prevalence is less well studied, but in general it is around 20% to 30% [2].

The aetiology of BV is still unclear but it is currently considered to be a synergistic polymicrobial syndrome, characterised by depletion of *Lactobacillus* spp and an intense increase (100- to 1,000-fold above normal levels) in vaginal anaerobic bacteria, including *Gardnerella vaginalis*, *Prevotella* spp, anaerobic gram-positive cocci, *Mobiluncus* spp, *Mycoplasma hominis* and *Atopobium vaginalis* [3, 4], leading to a replacement of lactobacilli and an increase in vaginal pH.

BV can often arise as a chronic or recurrent disease and the reason for recurrences is not well known. Based on fluorescence in situ hybridisation of vaginal biopsy specimens, Mendling et al [4] have demonstrated that BV is associated with the development of an adherent polymicrobial biofilm that is highly organised and contains abundant *Gardnerella vaginalis* on the vaginal epithelium. Bacterial biofilms have recently been associated with several recalcitrant infections (involving *Escherichia coli*, *Helicobacter pylori* and *Pseudomonas aeruginosa*) [5]. The biofilm enhances bacterial attachment to epithelial surfaces, allows bacteria to reach much higher concentrations than in luminal fluids, and prevents the drugs from reaching the bacteria, which reside in the film in a quiescent, latent state [6]. This observation might provide an explanation of the high rates of BV relapses [7, 8]. The presence of *Atopobium vaginae* strictly associated with *Gardnerella vaginalis* in the persistent and adherent bacterial biofilm has been confirmed [4], and seems to be the main reason for the failure of BV treatment [9]. Although some authors suggest that *Atopobium vaginae* may play a role in lowering the vaginal pH level through lactic acid production [10], Marconi et al have found that *Atopobium vaginae* content strongly increases if the vaginal pH is 4.9 or higher [11]. Therefore, they suggest that strains of this bacterium may have different capacities of lactic acid production but not to a significant level to safeguard the vaginal microflora.

Vaginal vitamin C is available as silicon-coated tablets containing 250 mg ascorbic acid. This formulation is able to release the vitamin over hours, allowing a statistically significant vaginal pH-lowering effect [12]. The efficacy of vaginal vitamin C in relieving BV signs and symptoms has been confirmed in two studies by Petersen et al [13, 14]. Moreover, Abbaspour et al have showed that the efficacy of vaginal vitamin C in BV treatment is non-inferior to that of standard local metronidazole gel therapy [15].

The main goal of BV therapy and, in particular, of BV prevention is to keep the vaginal pH at 4.5 or lower, in order to prevent the overgrowth of pathogens until the normal lactobacilli are re-established and able to maintain the pH themselves [16]. Anaerobes grow poorly at pH 4.5 or lower; the optimum pH for *Prevotella* spp and *Gardnerella vaginalis* growth is 6 - 7. In vitro studies show that the concentrations of these bacteria increase with increasing pH, but both are susceptible to low pH. An ideal way to manage recurrent BV would be to maintain the vaginal pH at 4.5 with a prophylactic treatment that is able to control the overgrowth of bacteria. In this context, a viable therapeutic agent could be ascorbic acid, as it may able to help re-establish and maintain the vaginal ecosystem.

Based on the above-mentioned considerations, the present study aimed to evaluate the potential effect of vitamin C, well known to have a vaginal pH-lowering effect, in the prophylaxis of BV relapses in patients who have been cured from a BV episode.

## Materials and Methods

### Ethics

The study was fully Good Clinical Practice (GCP) compliant and was reviewed and approved by all relevant Independent Ethics Committees. All patients provided written informed consent prior to entering the study.

### Design and treatments

This was a multicentre, randomised, double-blind, placebo-controlled, parallel-group clinical trial. The patient population, recruited in nine European sites located in Italy, Germany, Russia, Ukraine, Portugal and the Netherlands, consisted of 142 out-patient women, aged between 18 and 50 years, with history of recurrent episodes of BV defined as at least two acute episodes in the last 12 months. The BV diagnosis was based on the contemporary presence of at least three out of four Amsel criteria [17]: vaginal discharge, pH of vaginal fluid > 4.5, amine (fishy) odour of vaginal discharge after addition of 10% KOH (whiff test) and the presence of clue cells (> 20%) on microscopic examination.

Patients with diagnosis of human immunodeficiency virus (HIV) infection, gonorrhoea, *Treponema pallidum*, *Herpes genitalis*, trichomoniasis and/or candidiasis, or concomitantly treated with immunosuppressant, antibiotics, *Lactobacillus* preparations, acidifying agents, disinfectants, or vaginal douching were excluded.

Eligible women, cured (confirmed by the absence of three out of four Amsel criteria [17]) from an episode of BV by a course of either metronidazole or clindamycin, were randomly (1:1) assigned to receive 250 mg ascorbic acid vaginal tablets or placebo as prophylaxis for six monthly cycles, starting within 24 hours from the determination of ‘BV cure’. The patients applied one vaginal tablet once a day for 6 consecutive days per month after menses. The women were instructed to insert the tablets deep into the vagina at bedtime and were supplied with 250 mg ascorbic acid tablets or matching placebo.

The overall prophylaxis was set by six cycles of applications but, when the first BV relapse occurred (confirmed by the presence of three out of four Amsel criteria [17]), the patients were discontinued from the study.

### Assessments

Clinical visits were conducted at the end of each cycle, within 3 days of the end of the treatment, and 1 month after the end of the last cycle (final visit). The diagnosis of BV was documented by collecting two specimens of vaginal discharge: the first sample for microscopic examination (detection of clue cells) of the fresh smear and the second one for the whiff test. Moreover, BV signs and symptoms were recorded (itching, burning, dysuria, odour, erythema, oedema, and fissures) and scored by means of a four-point scale as follows: 0 = absent, 1 = mild, 2 = moderate and 3 = severe.

Vaginal pH measurements were performed by the patients themselves using a pH-measuring device, namely pHem-Alert^®^ (Gynex Corporation, USA) during each cycle: before and 12 hours after the first application, 12 hours after the last application and during the application-free period on days 10 and 15.

The primary efficacy variable of the study was the time to the first BV relapse diagnosed according to Amsel criteria [17]. Secondary end-points were the evaluation of the pH-lowering effect of vitamin C, the evaluation of the frequency of BV sign and symptoms during the prophylaxis period, global assessment of the acceptability of the product by investigators and patients and the evaluation of the safety of vitamin C. The acceptability and tolerability of the product were evaluated by the patient and the investigator, respectively, according to the following five-point scale: 1 = very good, 2 = good, 3 = fair, 4 = poor and 5 = intolerance.

### Statistical analysis

All randomised patients who fulfilled the inclusion/exclusion criteria and had received at least one dose of the study medication were included in the intention-to-treat (ITT) analysis. In case of premature discontinuation or missing data in completed patients, the last observation carried forward approach (LOCF) was used and the final available observation was considered as an end-point value. All enrolled patients were considered as the safety population. The sample size was determined based on the chi-square test and the power was 85% to detect an odds ratio (O.R.) of 0.26.

The randomisation list was computer-generated in blocks of four with treatments balanced (1:1) within blocks, by the Biometry Department of Medi Service s.r.l., Italy, using the SAS Package 8.2, validated routine from the Computerized System Validation Process. Variables of an ordinate classification and dichotomous type were tested by the chi-square test according to the Mantel-Hanszel extension.

The proportion of subjects free of BV relapse after three or six cycles of prophylaxis was calculated considering the time from randomisation to the occurrence of the first event or to the last visit if the event did not occur. The event-free survival curve (EFS) was estimated according to Kaplan-Meier and the EFS in the two treatment groups were compared by means of the log-rank test.

The Cox regression model was adopted to evaluate the effects of the therapeutic regimen, adjusting for different confounding or prognostic factors and to evaluate the impact on prognosis of these factors. Both baseline and time-dependent covariates were considered in the multivariable model. The pH-lowering effect and the vaginal signs and symptoms were analysed using the Mann-Whitney test for inter-treatment comparisons and the Friedman test for intra-treatment analysis. Investigators’ and subjects’ judgements on the acceptability of the product in the two treatment groups were compared using the Wilcoxon Rank Sum test.

All tests of hypotheses were performed using two-sided tests at a 5% significance level.

## Results

### Patient population

Overall, 142 women were randomised; 74 (52%) received ascorbic acid and the other 68 (48%) received placebo. The two groups were homogeneous for age, height and weight distribution. BV signs and symptoms (itching, burning, dysuria, odour, erythema, oedema and fissures) were fairly similar across treatment groups, as summarised in Table 1.

**Table 1:** Demographic and Baseline Characteristics in the Two Treatment Groups (Intention to Treat Population)

Variable	Vitamin C (n = 74)	Placebo (n = 68)	Total (n = 142)
Demographic data	74	68	142
Age (years) (mean ± SD)	31.2 ± 7.7	31.0 ± 7.2	31.1 ± 7.4
Body weight (kg) (mean ± SD)	61.0 ± 9.4	60.5 ± 8.4	60.8 ± 8.9
Height (cm) (mean ± SD)	165.3 ± 5.7	165.9 ± 6.2	165.6 ± 5.9
Itching (mean ± SD)	0.15 ± 0.4	0.16 ± 0.37	0.15 ± 0.38
Burning (mean ± SD)	0.14 ± 0.34	0.13 ± 0.38	0.13 ± 0.36
Dysuria (mean ± SD)	0.03 ± 0.16	0.04 ± 0.21	0.04 ± 0.19
Odour (mean ± SD)	0.09 ± 0.3	0.19 ± 0.43	0.14 ± 0.37
Erythema (mean ± SD)	0.01 ± 0.12	0.06 ± 0.24	0.04 ± 0.19
Oedema (mean ± SD)	0.00 ± 0.00	0.03 ± 0.17	0.01 ± 0.12
Fissures (mean ± SD)	0.00 ± 0.00	0.01 ± 0.12	0.01 ± 0.08

### Proportion of patients free of BV relapses

**Figure 1:**
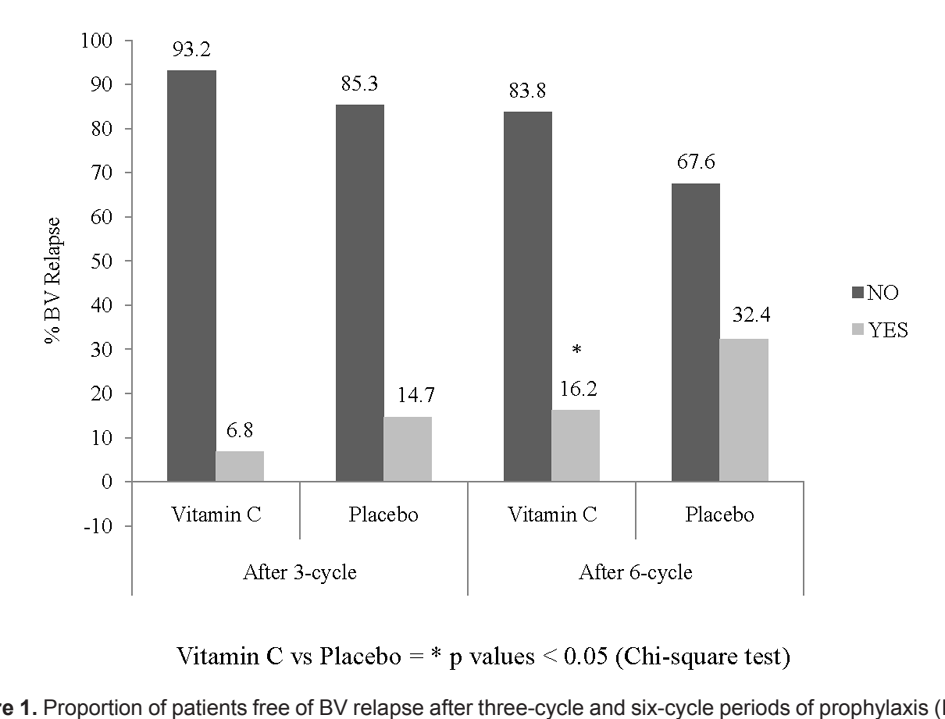
Proportion of patients free of BV relapse after three-cycle and six-cycle periods of prophylaxis (Intention-to-treat population, n = 142).

The proportion of women having a BV recurrence in the ITT population during the first 3 months was 6.8% in the vitamin C group (5/74) and 14.7% (10/68) in the placebo group, but the difference between the groups was not statistically significant. After 6 months of treatment, the recurrence rate was 16.2% in the vitamin C group compared with 32.4% in the placebo group (Fig. 1), which was statistically significant (P = 0.024, chi-square test). The results were confirmed by the O.R. that represents the risk of a recurrent episode of BV in the group treated with vitamin C in comparison to the placebo group. After six cycles of prophylaxis, the O.R. was 0.405 (95% confidence interval (CI) = 0.182 - 0.901). The result was statistically significant since the 95% CI of the O.R. did not include the value ‘1’.

**Figure 2:**
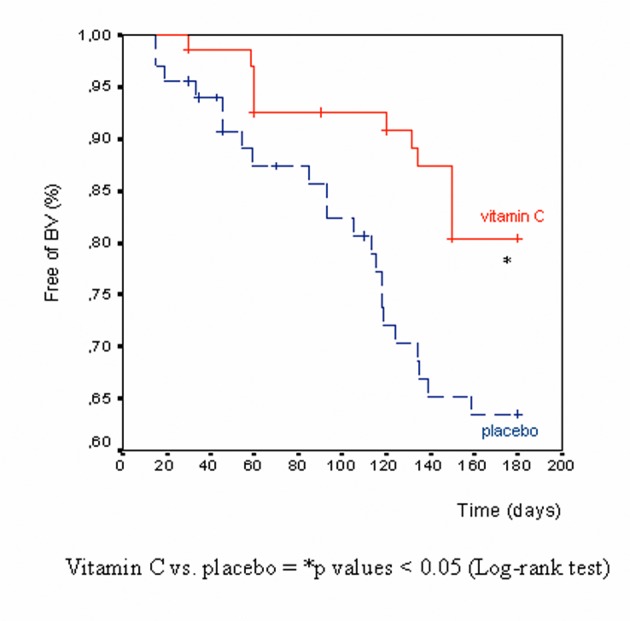
Kaplan-Meier survival analysis after six-cycle period of prophylaxis (Intention-to-treat population, n = 142).

The Kaplan-Meier survival analysis (Fig. 2), probability of being free of BV relapse in the two groups during the period of prophylaxis, became statistically significant starting from the 5-month time point (P = 0.039 vitamin C vs. placebo, log-rank test); the significant difference was maintained and increased at the 6-month evaluation (end of treatment, P = 0.029).

### Effect of prophylaxis on vaginal signs, symptoms and pH

**Figure 3:**
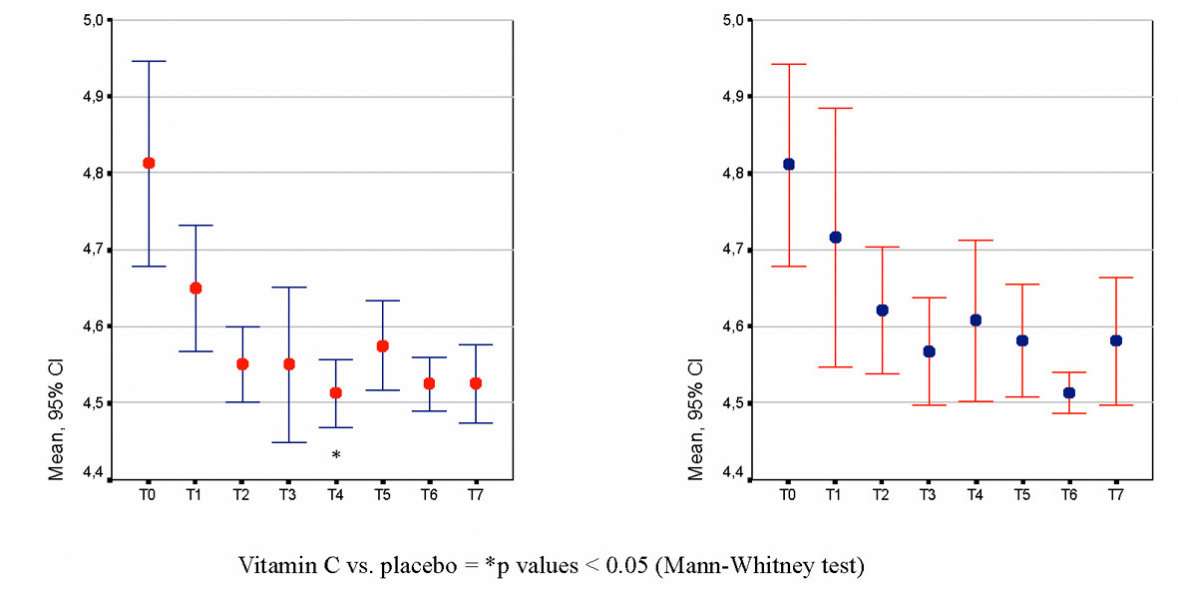
(a). Vaginal pH-lowering effect of vitamin C; (b): Vaginal pH-lowering effect of placebo.

BV signs and symptoms did not show any statistically significant differences through the treatment study period (from baseline to the follow-up visit), or in comparison of the treatment groups at the evaluated time points. The pH-lowering effect of vitamin C was detected after a prophylaxis period of 3 months, with a reduction of 3.8% compared with the baseline value and becoming statistically significant compared with placebo (Fig. 3) after four cycles of treatments (P = 0.032, Mann-Whitney test). The acceptability of treatment was judged as very good or good by 80.8% and 76.5% of investigators for the vitamin C and placebo groups, respectively, and by 76.7% of patients treated with vitamin C and by 75.0% of patients treated with placebo.

### Safety

The safety of vitamin C treatment was similar to placebo. The most common treatment-related adverse events (AEs) in both groups were local reactions such as itching, burning and skin irritation (n = 3 in the vitamin C group; n = 4 in the placebo group). All the AEs are showed in Table 2.

**Table 2:** Patients With at Least One AE (Safety Population)

Adverse event	Treatment group	
	Vitamin C (n = 74)	Placebo (n = 68)
Burning, itching, skin irritation	3 (4.0%)	4 (5.9%)
Candidiasis	1 (1.4%)	1 (1.5%)
Cystitis	-	1 (1.5%)
Nausea	-	1 (1.5%)
Bronchitis	1 (1.4%)	-
Major depression	-	1 (1.5%)
Flu	-	1 (1.5%)

Local tolerability was judged as very good or good by 75.3% of patients and 86.0% of investigators after vitamin C treatment and by 76.6% of patients and 82.0% of investigators after placebo.

## Discussion

A therapeutic approach in the treatment of BV relapse is to re-establish and maintain the physiological acidity of the vagina, as the growth of anaerobes and other faecal bacteria is inhibited by low pH. Attempts to achieve this via re-colonisation with exogenous lactobacilli have not been successful. Another, more accepted approach is to reduce vaginal pH, in order to create a negative environment for pathogen growth and to achieve long-lasting normalisation of vaginal flora using intravaginal ascorbic acid (vitamin C). The use of antibiotics may induce resistance in the pool of bacteria recognised to cause BV and, conversely, could affect the normal flora of lactobacilli [9], favouring recurrence within a few weeks in over 70% of women taking antibiotics for bacterial vaginosis [18]. Ascorbic acid (250 mg, in a silicone carrier that ensures prolonged action) plays a vital role in maintaining low vaginal pH values and enhances healing processes in the vaginal ecosystem - recolonisation with lactic acid bacteria. The mechanism of action is simple: through the lowering of vaginal pH to the physiological level of 3.8 - 4.5, anaerobic overgrowth is inhibited and the conditions for the re-growth of physiological lactobacilli flora are re-established.

The results of the present study show that 250 mg ascorbic acid vaginal tablets taken 6 days per month safely halves the risk of BV recurrence from 32.4% to 16.2% during a 6-month prophylactic treatment. The O.R. confirms that subjects treated with placebo had a doubled risk of BV recurrence compared with the group of subjects treated with vitamin C. Considering the time to the first BV relapse, treatment of at least five cycles is necessary in order to reduce, at a significant level, the risk of BV recurrence. As this was a prophylaxis study, in women who at the screening visit were healthy and who terminated the study in case of relapse, a between-treatment difference in clinical parameters was not expected. At the same time, differences in pH were not expected but conversely, a reduction in pH was noted for 3-month and 6-month treatment.

In conclusion, regular use of silicon-coated vitamin C (250 mg) tablets, after the standard antibiotic treatment for BV, protects women by reducing the risk of recurrence probably by re-establishing the normal lactobacilli flora that is able to maintain vaginal pH.

## Abbreviations

AE: adverse event
BV: bacterial vaginosis
EFS: event-free survival curve
GCP: good clinical practice
HIV: human immunodeficiency virus
ITT: intention-to-treat population
LOCF: last observation carried forward
O.R.: odds ratio
SD: standard deviation
